# Phosphodiesterase Type 5 Inhibitors and Visual Side Effects: A Narrative Review

**DOI:** 10.18502/jovr.v16i2.9088

**Published:** 2021-04-29

**Authors:** Francisco Barroso, João Crispim Ribeiro, Eduardo P. Miranda

**Affiliations:** ^1^Federal University of Ceara, Fortaleza, Ceara, Brazil; ^2^Christus University Center, Fortaleza, Ceara, Brazil

**Keywords:** Adverse Effects, Eye Manifestations, Physiological, Review, Sexual Dysfunction, Sildenafil Citrate

## Abstract

Phosphodiesterase type 5 inhibitors such as sildenafil citrate and tadalafil are well known for the treatment of erectile dysfunction. However, their use in the presence of pulmonary hypertension can cause ophthalmologic side effects, including non-arteritic optic ischemic neuropathy, chorioretinopathy, glaucoma, and optic atrophy. The present review aimed to identify these visual side effects and provide recommendations. We identified articles published from January 2000 to March 2019 on diseases arising from the management of sexual dysfunction in urology or pulmonary hypertension in pneumonia that could cause pathologic alterations in eye structure based on a literature search of the MEDLINE electronic database using keywords for the most common adverse effects and different kinds of phosphodiesterase 5 inhibitors. After applying the exclusion criteria, we selected 36 of the 77 articles initially identified to write the narrative review and added 20 additional articles to completely describe the pathological entities. Phosphodiesterase type 5 inhibitors can cause side effects in the eye including ocular surface abnormalities, increased intraocular pressure and glaucoma, uveitis, non-arteritic ischemic neuropathy, chorioretinopathy, retinal occlusion, and visual field changes. There is an increased need for well-performed studies to better understand these side effects, which are common due to the wide use of sildenafil.

##  INTRODUCTION

Erectile dysfunction (ED) is defined as a persistent inability to achieve and/or maintain an erection to allow satisfactory sexual relations. It is a prevalent condition, with over 18 million men affected worldwide. ED affects an estimated 9.1% of men aged 40–49 years, 15.2% of men aged 50–59 years, 29.4% of men aged 60–69 years, and 54.9% of men older than 70 years.^[1]^ The first line of treatment for ED is phosphodiesterase type 5 inhibitors (PDE5i). This drug was first developed to treat pulmonary hypertension and muscle spasms; however, its most notable side effect, prolonged erection, soon became the focus of its use in clinical practice. In 1998, the Food and Drug Administration (FDA) approved its use as a clinically effective and safe treatment for ED.^[2]^

There are 11 types of PDEs, all of which function to degrade cyclic adenosine monophosphate (AMPc) to adenosine monophosphate (AMP) and GMPc to guanosine monophosphate (GMP).^[3]^ Sildenafil citrate (SC), a selective inhibitor of PDE5i present in the cavernous bodies in the penis, is responsible for smooth muscle relaxation and, consequently, inducing an erection.^[1]^ PDE5i can cross the blood–brain and blood–retinal barriers and may partially inhibit the PDE 6 enzyme present in the retina, with significant dose-dependent changes in photoreceptors and the optical nerve.^[4,5,6]^ The main contraindication for the use of PDE5i is its concomitant use with nitrates, which can lead to hypotension and ischemic events.^[4]^

The main reported side effects of PDE5i are headaches, dizziness, blushing, nasal congestion, dyspepsia, and visual changes. The most common visual side effects are photophobia, cyanopsia, and haze. Most of the visual effects can be reversed weeks after stopping use of the medication.^[3]^ Other ophthalmologic disturbances described in the literature include keratitis, ocular surface abnormalities, chorioretinopathy, vessel occlusion, retinal detachment, and optic neuropathy.^[1,4,5,7]^

Considering the wide use of PDE5i, clinicians should know its side effects profile. This study reviewed the most common ocular effects related to the use of PDE5i.

##  METHODS

A literature search was conducted through the MEDLINE online electronic database for articles written in English published from January 2000 to March 2019. The reason to delimitate the research to this period was to evaluate recent developments regarding PDE5i and their side effects. Thus, this review aimed to perform a current, but thorough, analysis of published articles. We used the following descriptors in sequence, all of which had to be present in the title of the article: (1) Keratitis OR (2) Surface Abnormalities OR (3) Chorioretinopathy OR (4) Ocular OR (5) Intraocular Pressure OR (6) Glaucoma OR (7) Non-arteritic Anterior Ischemic Optic Neuropathy OR (8) Retinal Occlusion OR (9) Optic Atrophy OR (10) Visual Changes OR (11) Electroretinography OR (12) Uveitis OR (13) Retinal AND (14) Sildenafil AND (15) Phosphodiesterase 5 Inhibitors AND (16) Tadalafil AND (17) Viagra AND (18) Vardenafil AND (19) Avanafil AND (20) Udenafil AND (21) Mirodenafil AND (22) Lodenafil AND 2000/01/01: 2019/03/31. The keywords from 1 to 13 were searched again with AND 14 added; the same process was performed to add keywords 15 to 22.

##  RESULTS

**Figure 1:**
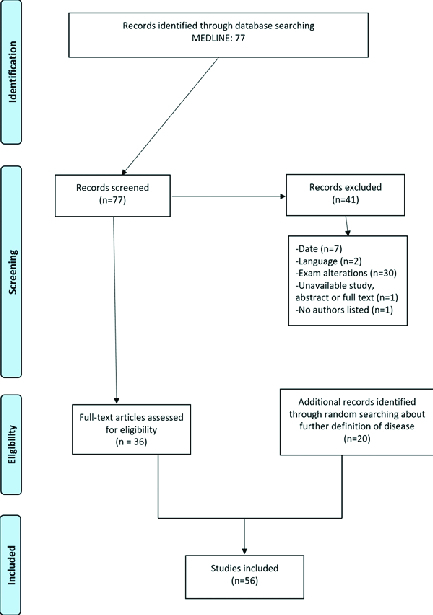
Inclusion and exclusion criteria.

We selected 36 of the 77 articles initially identified for the literature review, including both clinical and experimental studies. We also included 20 additional articles to completely describe the pathological entities [Figure 1]. We analyzed the articles for the inclusion and exclusion criteria. The inclusion criteria were articles that had in their titles at least one combination of the descriptors in the search strategy, publications written in the English language, and relevant content in the article regarding the research topic. The exclusion criteria were articles that had in the title or abstract nonophthalmic PDE adverse effects related to PDE5i use; reviews and book chapters; non-original studies including editorials, exam alteration studies, preface and brief communication; duplicate articles, articles written in a language other than English; and unavailable study abstract or full text. We added two studies related to non-arteritic anterior ischemic optic neuropathy (NAION) since our search method identified few studies. Furthermore, we also included 13 letters to editors that described important side effects. We did not find optic atrophy studies related to our topic; similarly, no studies on mirodenafil, udenafil, or lodenafil were identified. Each retrieved article was carefully read and checked for relevance before being included in the review [Figure 1].

**Table 1:** The best studies for each category


**Finding**	**Authors**	**Concise Methods**	**Brief Outcomes**	**Strength of Evidence**
**Chart 1: Studies Related to Intraocular Pressure Alterations**				
Increased intraocular pressure	Nazari et al. 2017^[20]^	-110 patients -Weekly sildenafil of 50mg average dose after 3 months	-Significantly differences between intraocular pressure values -p=0,003	-Grade B
	Wirostko et al. 2012^[15]^	-277 adults	- Changes in Intraocular Pressure mean showed in 95% confidence interval (CI)	-Grade B
	Ermis et al. 2004^[25]^	-28 male volunteers	-No significantly changes in intraocular pressure -p=0.37	-Grade B
	Dündar et al. 2001^[26]^	-14 healthy male volunteers	- No significantly changes in intraocular pressure	Grade B
No Increased intraocular pressure	Yajima et al. 2000^[24]^	-Study 1with 16 subjects and study 2with 48 subjects	-No major changes	- Grade B
**Chart 2: Other Surface Abnormalities**				
**Finding**	**Authors**	**Concise Methods**	**Brief Outcomes**	**Strength of Evidence**
Surface abnormalities	Matieli et al 2016^[8]^	-Case control with eyes in case group and 13 eyes in control group	-28 eyes (70%) using sildenafil, while only 13 eyes (35%) in the group control	- Grade B
Chart 3: Keratitis				
Finding	Authors	Concise Methods	Brief Outcomes	Strength of Evidence
-Keratitis	Matieli et al 2016^[8]^	-Case control with 20 patients	-One-third of individuals	- Grade B
**Chart 4: Chorioretinopathy**				
**Finding**	**Authors**	**Concise Methods**	**Brief Outcomes**	**Strength of Evidence**
Central Serous Chorioretinopathy	Turkuc et al 2013^[11]^	-43 patients treated	-Sildenafil citrate did not lead to Central Serous Chorioretinopathy	-Grade B
**Chart 5: Uveitis**				
**Finding**	**Authors**	**Concise Methods**	**Brief Outcomes**	**Strength of Evidence**
Uveitis	French et al^[12]^	-Case report	- 6 episodes of uveitis	- Grade C
	Pomeranz et al^[34]^	-Literature review	- 40 case report studies reported	- Grade C
	Galvez-Ruiz et al^[36]^	-Case series	- 10 patients who after regular intake of Sildenafil presented in 1 eye	- Grade C
NAION	Gedik et al^[37]^	-Case report	- 36-year-old male who presented blurry vision in his left eye after 100 mg Sildenafil intake	- Grade C
	Hafidi et al^[38]^	- Case report	- 40-year old man with vision loss after 100 mg Sildenafil for two consecutive days	- Grade C
	Pomeranz et al^[39]^	- Case series	- 5 patients presented after ingestion of 50 to 100 mg of Sildenafil	- Grade C
	Pomeranz et al^[40]^	-Case series	- 6 of 7 patients who took Sildenafil presented vision loss within 24 hours	- Grade C
**Chart 7: Retinal Vessel Occlusion**				
**Finding**	**Authors**	**Concise Methods**	**Brief Outcomes**	**Strength of Evidence**
Supratemporal retinal artery branch occlusion	Tripathi et al^[33]^	-Case report	69-year-old male who presented with sudden painless vision loss after two days of Sildenafil 100 mg intake	-Grade C
Cilioretinal artery occlusion	Hafidi et al^[38]^	-Case report	-40-year-old male who presented with acute vision loss after 2 days of Sildenafil 100 mg intake	-Grade C
	Murthy et al^[47]^	-Case report	-Bilateral concurrent cilioretinal artery occlusion in a 37-year-old female patient after once daily for four weeks	-Grade C
Cilioretinal vessel occlusion	Pinto et al^[49]^	-Case report	-36-year-old male presented with cilioretinal vessel occlusion	-Grade C
**Chart 8: Visual Change**				
**Finding**	**Authors**	**Concise Methods**	**Brief Outcomes**	**Strength of Evidence**
-Color discrimination alterations	Cordell et al^[56]^	-Clinical trial	- Any significant evidence after compared Sildenafil intake for 6 months daily	-Grade A

We divided the visual side effects into the following categories: keratitis, other surface abnormalities, chorioretinopathy, intraocular pressure and glaucoma, uveitis, NAION, and retinal occlusion. We described each category in different sections; namely, epidemiology and pathophysiology, clinical presentation, literature review, and final statement recommendation.

##  DISCUSSION

### Keratitis

#### Epidemiology and pathophysiology

In less developed countries, infectious keratitis affects about 10% of the population. Eye injury during agricultural work may predispose infectious agents to cause keratitis. In developed countries, the main cause of this condition is the use of contact lenses.

#### Clinical presentation

Keratitis presents intense itching accompanied by redness, pain, photophobia, red eye, and a “gritty” sensation.^[4]^

#### Literature review

We did not find any association between PDE5i use for ED and keratitis. However, a case–control study that evaluated ocular toxicity in patients with pulmonary arterial hypertension (PAH) reported a higher incidence of keratitis following chronic SC use. Twenty patients in each group were administered SC from 1 to 60 months at daily doses of 60 mg, except for two patients treated with 120 mg per day. One-third of PAH individuals using SC had severe noninfectious bilateral keratitis. Such patients were not contact lens users. A multivariate analysis using a linear regression model showed that dry eye keratitis was significantly associated with SC use. The patients had no other risk factors for dry eye, suggesting that SC use was possibly the main cause of keratitis in these patients.^[8]^

#### Final statement recommendation

Patients who will start chronic PDE5i use and have complaints of dry eye or who use contact lenses should be counseled about an increased risk of keratitis. Referral for routine ophthalmic assessment and use of Preservative-free tear substitutes should be considered.^[8]^

### Other Surface Abnormalities 

#### Epidemiology and pathophysiology

Dry eye disease (DED) is the most common disorder related to surface abnormalities and has been associated with a wide range of traits, including systemic and metabolic conditions.

#### Clinical presentation

Dryness, burning, itching, watering, stickiness, and crustiness.^[9]^

#### Literature review

Only one article was identified related to this topic. In the same case–control study that evaluated ocular toxicity in PAH patients using SC, all participants presented ocular motility, contrast sensitivity, color vision, Schirmer test, intraocular pressure (IOP), and optical coherence tomography (OCT) within the normal ranges. At least one of these other abnormalities of the ocular surface (tarsal gland dysfunction, conjunctival hyperemia, cornea verticillata, and decreased rupture time) were present in 28 eyes (70%) using SC, compared to only 13 eyes (35%) in the control group. Considering the presence of at least one abnormality in biomicroscopy, SC users (40 eyes of 20 patients) had a statistically higher occurrence of anterior superficial anomalies (Fisher's test *p* = 0.012).^[8]^

#### Final statement recommendation

Patients with PAH chronically treated with SC should undergo routine ophthalmologic evaluations to identify ocular surface abnormalities.^[8]^

### Chorioretinopathy

#### Epidemiology and pathophysiology

Central serous chorioretinopathy (CSC) typically affects men and women between their third and sixth decades of life.^[11]^ The major risk factors are the state of refraction, systemic hypertension, male sex, older age, black ethnicity, and use of corticosteroids.^[10]^

#### Clinical presentation

CSC presents as a painless dark, blurred, distorted, dimmed area in the central vision.^[11]^

#### Literature review

Two articles on this topic found no association between SC use and CSC. In a prospective study, 43 patients with a mean age of 49.1 years (range 28–67) were treated for ED with SC (50 mg two to three times per week for one month). The patients were evaluated in the first week and at the end of treatment. Macular thickness and volume assessments with OCT did not differ significantly in the central and parafoveal areas (*p* > 0.05). The study concluded that SC in therapeutic doses did not lead to CSC and visual abnormality.^[11]^ French et al^[12]^ studied 577 men aged 59 years and younger with newly diagnosed CSC. To avoid confounding factors, this study did not include individuals with age-related macular degeneration and history of corticosteroid use. The authors found that 111 patients (19.2%) were prescribed PDE5i before CSC onset, and most (>99%) were administered SC doses of 100 mg or more [odds ratio: 1.05, 95% confidence interval (CI): 0.74–1.22].^[12]^ In contrast, Gordon-Bennett et al^[13]^ reported an inkblot appearance near the fovea in a 51-year-old male patient 24 hr following the ingestion of more than 20 mg tadalafil.

#### Final statement recommendation

Patients who are about to start ED treatment with PDE5i should be advised to consult an ophthalmologist in case they experience vision problems.^[11]^

### Intraocular Pressure and Glaucoma 

#### Epidemiology and pathophysiology

The global prevalence of glaucoma was over 57 million individuals in 2015 and is expected to increase to over 65 and 111 million individuals by 2020 and 2040, respectively. Angle closure and reduction of ocular blood flow are additional mechanisms for the development of glaucoma, which can be triggered by the systemic use of vasodilators, including some PDE5i.^[14,15,16,17]^

#### Clinical presentation

In primary open-angle glaucoma, which is the most common type of glaucoma, the patient is usually oligosymptomatic and visual changes are present only in advanced stages. In acute-angle glaucoma, sudden visual pain followed by nausea and vomiting is usually the initial presentation.^[18]^

#### Literature review

Nine articles discussed this topic. Some authors reported increased IOP after SC use.^[19]^ Nazari et al^[20]^ reported increased mean IOP after three months in 110 patients aged 42–60 years with weekly SC doses of 25–100 mg (*p* = 0.002).^[20]^ Furthermore, Chen et al^[21]^ evaluated the prevalence of self-reported glaucoma in a telephone-based interview in men aged over 40 years. They found that 482 of 7,081 participants reported a diagnosis of glaucoma and the use of SC in the previous year. However, the dose and frequency of PDE5i intake were not investigated.^[21]^ Gerometta et al^[22]^ studied nine healthy male and female volunteers aged 18–74 years, in which the IOP increased 60 min after SC administration (*p* < 0.005) and returned to the control values within 2 hr.^[22]^ Gerometta et al^[23]^ also demonstrated increased IOP in 21 normal sheep after ingestion of both SC (50 and 100 mg) and tadalafil. Other research has shown no association between SC use and IOP increase. Yajima et al^[24]^ did not detect any major changes in IOP 24 hr after the oral administration of 10–150 mg SC I. Furthermore, Wirostko et al^[15]^ reported no changes in IOP among 277 adults with idiopathic PAH in a double-blind study using oral SC (20, 40 or 80 mg) or placebo (1:1:1:1) three times daily for 12 weeks.^[15]^ Ermis et al^[25]^ failed to show any significant effects of SC use on IOP in 28 healthy volunteers with a mean age of 51 years receiving SC (50 and 100 mg).^[25]^ Moreover, in a double-blind, randomized, placebo-controlled, crossover study, Grunwald et al^[16]^ reported no significant acute changes on IOP after a single oral dose of SC l 100 mg in 15 subjects aged 63 ± 14 years with bilateral chronic open-angle glaucoma.^[16]^ Dündar et al^[26]^ also showed no change in IOP after the administration of a single oral dose of 50 mg SC to 14 healthy male volunteers tested immediately before and 1 hr after SC administration.^[26]^

#### Final statement recommendation

There is a lack of prospective randomized trials to determine whether there is a true causal association between PDE5i use and elevation of IOP leading to glaucoma development or deterioration. However, it is advisable to monitor IOP periodically in high-risk patients^[20]^ [Table 1].

### Uveitis

#### Epidemiology and pathophysiology

The prevalence of idiopathic uveitis is similar in studies from different countries. Although the understanding of the pathogenesis of uveitis is evolving, approximately 24–55% of patients with uveitis have idiopathic or undifferentiated uveitis.^[27]^

#### Clinical presentation

Uveitis is an intraocular inflammation due to an inflammation of the uvea presenting as redness and swelling of the uvea.^[28]^

#### Literature review

Only one article discussed this topic. The association of PDE5i and the onset of uveitis is limited to one case report, which described a 38-year-old man diagnosed with Behçet's disease 15 years prior and without any occurrence of uveitis for the past 12 years. After starting SC for ED, he developed recurrent posterior uveitis in his left eye. The uveitis episode started after the second or third dose of SC, with a total of six episodes.^[29]^

#### Final statement recommendation

Patients with ED who plan to start PDE5i should be informed of the risk for ocular side effects. If a recurrence of ocular symptoms is observed concomitant to PDE5i intake, patients should be informed to stop treatment.^[29]^

### NAION

#### Epidemiology and pathophysiology

The annual incidence of NAION is 1 in 10,000 cases and affects men and women equally. It is more common in Caucasians than in African, American, and Hispanic descendants. NAION is a multifactorial disorder^[30]^ and is believed to be related to vascular insufficiency in the optic nerve head. The condition is also associated with short posterior ciliary artery dysfunction. However, the mechanisms related to the vasculopathy and ischemia remain unclear and there is no consensus regarding the possible associations between NAION and PDE5i.^[31,32]^

#### Clinical presentation

The primary presentation includes acute, painless, unilateral visual loss, which confers an increased risk of contralateral vision loss. The condition may worsen over hours or days, with optic disc edema and an afferent pupillary defect. Altitudinal defects are a more common presentation in the visual field.^[16,33]^

#### Literature review

In their literature review, Pomeranz et al^[34]^ described 40 case reports of NAION. Posterior ischemic optic neuropathy was also related to SC use in some letters to the editor.^[30,35]^ Galvez-Ruiz et al^[36]^ reported a series of 10 patients who presented with NAION with routine exposure to SC (>2–3 times per week) during the weeks and months before the ocular ischemia. NAION was diagnosed based on clinical presentations, fundus features consistent with NAION, and exclusion of other possible etiologies. Half of the patients in the study had a primary episode of unilateral ischemic optic neuropathy. Despite the initial adverse event (first episode of NAION), all patients continued to use the medication and developed a second episode of NAION in the contralateral eye. Only 1 of the 10 patients presented with bilateral simultaneous NAION.^[36]^ Another report described a 36-year-old male patient presenting with blurred vision in his left eye and afferent pupillary defect after the ingestion of 100 mg of SC. Funduscopy revealed hyperemia and edema in the lower portion of the left optic disc.^[37]^ Hafidi et al^[38]^ reported an acute unilateral visual loss in a previously healthy 40-year-old man after ingestion of 100 mg SC for two consecutive days before the onset of this visual symptom. In this particular case, SC was associated with NAION and cilioretinal and central retinal vein occlusion (CRVO).^[38]^ Pomeranz et al^[39]^ (2002) reported on five patients ranging in age from 42 to 69 years after SC ingestion. They demonstrated the characteristic findings of NAION, including altitudinal visual field loss and associated optic disc and/or peripapillary hemorrhages. Pomeranz et al^[39]^ later reported on seven patients aged 50–69 years who had typical symptoms of NAION within 36 hr after the ingestion of SC for ED.^[37,40]^ However, in a cohort of 8,893 patients prescribed SC by their primary care physicians in England between April and June 1999, NAION was reported only in a 61-year-old patient who had other risk factors for NAION; thus, it was not possible to confirm any association between SC and NAION.^[40,41]^ However, Peter et al^[42]^ reported an NAION case in a patient with no known vasculopathy risk factors.

#### Final statement recommendation

A history of NAION should be an absolute contraindication to PDE5i therapy^[43]^ and patients who are at higher risk for NAION (such as small cup-to-disc ratio and systemic arterial hypertension) should be counseled and undergo ophthalmologic assessments.^[35,38,44]^

### Retinal Vessel Occlusion 

#### Epidemiology and pathophysiology

Central retinal artery occlusion (CRAO) occurs in an estimated 1 in 100,000 individuals in the general population and accounts for 1 in 10,000 ophthalmological outpatient visits.^[45]^ An embolism is the most common cause of CRAO.^[46]^

#### Clinical presentation

CRAO appears as a sudden, catastrophic visual loss.^[46]^

#### Literature review

Tripathi et al^[33]^ reported a case of a 69-year-old man presenting with a sudden painless loss of vision in the left eye two days previously. Fundus examination of the right eye was normal whereas the left eye showed a supratemporal branch retinal artery occlusion. As the patient did not have any of the risk factors for arterial occlusion, a more detailed history was sought. The patient indicated that he had taken SC (100 mg) a few hours before he experienced the loss of vision in his left eye.^[33]^ Murthy et al^[47]^ reported a case of bilateral concurrent CRAO in a 37-year-old female African–American patient with sickle cell disease and PAH. She had been treated with tadalafil (40 mg) once daily for four weeks. Fluorescein angiography (FA) revealed delayed transit time with areas of blocked fluorescence due to retinal edema,^[48]^ and spectral-domain OCT revealed inner retinal edema in the macula.^[47]^ Hafidi et al^[38]^ reported a case of CRAO and CRVO observed by OCT and FA. The 40-year-old male patient was previously healthy and presented with acute visual loss of the left eye after two days of 100 mg SC use.^[47]^ Pinto et al^[49]^ reported a case of a 36-year-old man with chronic renal failure who was diagnosed with CRVO by FA after ingesting 100 mg SC.

#### Final statement recommendation

Acute and severe complaints should alert clinicians to CRAO. Patients with increased risks for thromboembolic events should be counseled about CRAO. Despite the anecdotal cases, it is not possible to establish a link between PDE5i use and CRAO.^[47,50]^

### Visual Changes 

#### Epidemiology and pathophysiology

Most cases of visual changes after PDE5i are believed to be dose dependent and lead to mild alterations in the visual system. These alterations lead to complaints of blue-tinged vision and increased brightness perception.^[51]^ SC also inhibits phosphodiesterase type 6 (PDE6),^[52]^ an essential enzyme involved in the activation and modulation of the phototransduction cascade, impairing the ability of PDE6 to shorten the time of integration between the visual system and light level.^[53]^ The sensitivity of SC for PDE6 isoenzymes is thought to be the basis for the reduced electrophysiological response on electroretinograms (ERG) in patients prescribed 50 mg SC, as well as the transient color vision abnormalities more likely to be observed with higher doses of SC.^[54]^

#### Clinical presentation

Patients report changes related to color perception.^[55]^

#### Literature review

In a comparative case series study, Matieli et al^[8]^ analyzed the visual effects of SC use for PAH by assessing symptoms such as bluish vision, photophobia, and green or blue or yellow patterns on Farnsworth D15-Color Test Saturated Panels. SC was used from 1 to 60 months. The SC user and control groups included 17 women each. One-third of the treated group showed severe bilateral keratitis. In contrast, Cordell et al^[56]^ randomized 244 subjects to receive tadalafil (5 mg, 85 patients), SC (50 mg, 77 patients), or placebo (82 patients) daily for six months. No significant differences in the visual function of color discrimination by the Farnsworth–Munsell 100-Hue Color Vision Test were observed between the treatment and placebo groups.^[56]^

#### Final statement recommendation

Despite its low occurrence, patients should be counseled about transient color changes associated with PDE5i use for ED or PAH.^[8]^

##  SUMMARY

PDE5i are one of the most prescribed drugs worldwide, with a very good safety profile. However, NAION, chorioretinopathy, and color changes are important issues reported in the literature. Although these visual side effects have been reported in patients using PDE5i, there is not enough evidence to prove a causal association. Given the low incidence of such side effects, these should not preclude the usual indications for PDE5i. However, patients with histories of ophthalmologic conditions or optic nerve issues or those at high risk for any eye disease require specific clearance before the commencement of therapy.

##  Financial Support and Sponsorship

Nil.

##  Conflicts of Interest

There is no conflict of interest.
